# Editorial: Feline tumors of nervous system

**DOI:** 10.3389/fvets.2023.1200687

**Published:** 2023-05-05

**Authors:** Maria Teresa Mandara, Martí Pumarola

**Affiliations:** ^1^Department of Veterinary Medicine, University of Perugia, Perugia, Italy; ^2^Department of Animal Medicine and Surgery, Universitat Autònoma de Barcelona, Barcelona, Spain

**Keywords:** cat, embryonal tumor, lymphoma, magnetic resonance, nervous system, schwannoma, immunophenotype

Cats and dogs share human environments and habits which in the last decades have extended life expectancy in pets as in man, and they will do more in the next (1). This is why we have no doubts to assess that the cat develops tumors with an incidence similar to the dog, except for species-related predisposition or viral oncogenetic infections. If this assumption is correct and probably no far from the true also for tumors of the nervous system, a large number of nervous system tumors still escape from specialist investigations, diagnosis, therapeutic approaches and, finally, from studies. Considering the number of papers published about feline tumors of nervous system in 2022, in PubMed website we find only nine papers vs. more than 30 papers for the dog. Additionally, more difficult is to detect wide case series studies also for tumors with high incidence in this species, as it is lymphoma. Based on these premises, in this Research Topic we wanted to promote and collect researches on this subject, and the obtained results are consistent with the aim enough to be proud to have contributed by increasing the literature on feline tumors of the nervous system of about 50% for the last year.

This Research Topic includes two case reports and two original researches. The high-quality description and the originality of the case reports confirm how much medicine science and specifically feline medicine need them to add details and information in a broader context or to create a new context of focus. I owe it to Dr. Robert J. Higgins of UC of Davis in California (USA), who left us just over a year ago, to have taught us, when we were young people entering the world of research, to not neglect the value of case reports since, as he said, “*their importance in knowledge advancement is all the more recognized, the less we know about*.” Contrarily to the Editorial policy of many scientific journals, we are also pleased that Frontiers included Case Reports.

That's how today we know that the cat can develop neuroblastoma-like schwannoma and Embryonal Tumor with Abundant Neuropil and True Rosettes (ETANTR), thanks to detailed histological descriptions, in the first case also supported by electron microscopy analysis, and wide immunohistochemistry investigations in both. In the paper of Chen et al. the identification of pseudo-Homer-Wright rosettes characterizing the neuroblastoma-like schwannoma is really an intriguing issue. In this study negative immunolabelling for synaptophysin definitely excluded typical figures of neuroblastoma, while periaxin (2–4) and SOX-10 (5) have proven their selves as useful markers to identify the Schwann cells in the cat, further supported by strong reaction to laminin, in the controversial field of differential diagnoses to peripheral nerve sheath tumors. However, if Mahjoub et al. (6) assumed the pseudo-rosette pattern should be distinguished from Verocay bodies based on different arrangement of the neoplastic Schwann cells (horizontal rows vs. pseudo-circular alignment of palisaded nuclei), we think that this difference does not definitely support they really represent two different morphological patterns, although the former is collagen-rich, the latter no. Therefore, further studies on this morphological variant are welcome also to define its potential biological meaning in the tumor behavior.

Again, human neuropathology shows us many histological variants of tumors of the nervous system which, far from having to simply be assumed in veterinary classification, represent a continuous pressure for comparative and behavioral studies in veterinary neuropathology. Embryonal Tumor with Multilayered Rosettes (ETMR) is a clear example of this, encouraging us to reconsider the large group of non-cerebellar Primitive Neuroectodermal Tumors in pets. The paper by Foiani et al. rises up a couple of questions: (1) based on the only case of feline embryonal tumor of the central nervous system (CNS) reported in the literature, can really this species be assumed as not predisposed to juvenile CNS tumor? (2) what should “juvenile” mean in cats? Less than 2 years or < 1 year? Or rather generically < 5 years, considering five as the line beyond which the incidence of neoplastic diseases tends to increase in pets? Whatever our answers are, as from today ETANTR must be included in differential diagnosis of non-cerebellar PNET in cats and domestic animals, and classification of CNS tumors should be updated in veterinary medicine. Of course, we are long way from made diagnosis by genetic investigations as in humans, but we think that for juvenile tumors a greater effort may be worth making in veterinary oncology. As for the feline lymphoma (Mandara et al.), one of the most common tumors of the nervous system, the anatomical patterns have been confirmed to be numerous, as in the past review (7), and they call for different clinical weights and approaches. Albeit a long way still to go in characterizing lymphoma of the nervous system to therapeutic and prognostic purposes, the authors collected sufficient data to strongly suggest that extra-axial lymphoma, except for leptomeningeal lymphomatosis, mainly expresses B-cell phenotype. Along with the highest surgical accessibility to extra-axial tumors, this result further supports the most favorable prognosis of this anatomical pattern compared to the intra-axial counterpart. And remember not to overlook lymphoma of the nervous system also as a primary neoplastic disease and the typical involvement of the optic chiasm in the cranial cavity. In the second work related with feline lymphoma affecting the spinal cord (Lorenzo et al.) it is worth appreciating the collaborative effort among clinic, imaging, surgery, and neuropathology made to add more information about the most common spinal cord tumor in this species. Collecting data in all these fields from each case is not always an easy task. Combining localization of tumors, clinical findings, cerebrospinal fluid analysis, magnetic resonance imaging characteristics and their possible correlations with histopathological findings, especially for the presence of necrosis, have provided useful markers for both final diagnosis and prognosis of the cases.

Upon completion of this editorial project, we want to thank all the contributing authors since new interesting and updated information on feline tumors of the nervous system have been provided for use by scientific community and veterinary neurologists, and their daily work progress.

## Author contributions

MM wrote the body of the editorial. MP wrote part of comments and added references. Both authors contributed to the article and approved the submitted version.
